# From damage response to action potentials: early evolution of neural and contractile modules in stem eukaryotes

**DOI:** 10.1098/rstb.2015.0043

**Published:** 2016-01-05

**Authors:** Thibaut Brunet, Detlev Arendt

**Affiliations:** European Molecular Biology Laboratory, Developmental Biology Unit, Heidelberg 69012, Germany

**Keywords:** electrophysiology, evo-devo, action potentials, musculature, nervous systems, evolution

## Abstract

Eukaryotic cells convert external stimuli into membrane depolarization, which in turn triggers effector responses such as secretion and contraction. Here, we put forward an evolutionary hypothesis for the origin of the depolarization–contraction–secretion (DCS) coupling, the functional core of animal neuromuscular circuits. We propose that DCS coupling evolved in unicellular stem eukaryotes as part of an ‘emergency response’ to calcium influx upon membrane rupture. We detail how this initial response was subsequently modified into an ancient mechanosensory–effector arc, present in the last eukaryotic common ancestor, which enabled contractile amoeboid movement that is widespread in extant eukaryotes. Elaborating on calcium-triggered membrane depolarization, we reason that the first action potentials evolved alongside the membrane of sensory-motile cilia, with the first voltage-sensitive sodium/calcium channels (Na_v_/Ca_v_) enabling a fast and coordinated response of the entire cilium to mechanosensory stimuli. From the cilium, action potentials then spread across the entire cell, enabling global cellular responses such as concerted contraction in several independent eukaryote lineages. In animals, this process led to the invention of mechanosensory contractile cells. These gave rise to mechanosensory receptor cells, neurons and muscle cells by division of labour and can be regarded as the founder cell type of the nervous system.

All the essential problems of living organisms are already solved in the one-celled … protozoan and these are only elaborated in man or the other multicellular animals.

G. G. Simpson, *The Meaning of Evolution*, 1941. [1]

## 1. Introduction

The intracellular composition of all living cells differs radically from that of extracellular fluids: the cytoplasm is richer in potassium, poorer in sodium—and, in particular, much poorer in calcium (which does not exceed 10^−7^ M in the resting cell but reaches 10^−3^ and 10^−2^ M in blood and seawater, respectively) [2,3]. The peculiar chemistry of the cytoplasm is often assumed to reflect the environment of the first cells [4,5]. Indeed, based on their reconstituted membrane composition (rich in simple single-chain lipids), primitive cells were probably leaky to small molecules—their intracellular ionic balance thus necessarily matching the one of their environment [6,7]. One such possible environment could have been geothermal fields [5]. The composition of the primordial ocean itself is debated [8], but it could have been calcium-rich from the very beginning, or calcium could have accumulated as recently as 1.5 billion years ago [9]. In any case, the presence of abundant extracellular calcium poses a special challenge to cellular life, as a high quantity of intracellular calcium is highly toxic to all living cells. One key reason is that energetic metabolism is universally phosphate-based (e.g. ATP hydrolysis and synthesis of nucleic acids release phosphate ions), but calcium readily forms insoluble precipitates with phosphate [2,3,10].

The evolving discrepancy between intracellular and extracellular chemistry forced concomitant adaptations of living cells. No known cell has altered its cytoplasmic composition to match modern environmental conditions but, instead, ways to maintain the old cytoplasmic chemistry in the new environment have evolved. The most prominent are active sodium and calcium efflux pumps: Na^+^ and Ca^2+^ efflux ATPases are widespread in both eukaryotes and prokaryotes, and are probable ancestral features of all living cells [11–19]. Another shared strategy is concentration of calcium in specialized storage spaces, both intracellular [20–24] and extracellular, like cell walls or skeletal structures [10,25,26]. This discrepancy between the intracellular and extracellular medium has two further consequences: the necessity to isolate the cell content—by enclosing it in tight membranes, and quickly repairing any wound; and the necessity to maintain the transmembrane voltage close to its setpoint—as required for the integrity of membrane protein structure [27]. These two protective mechanisms probably evolved in stem eukaryotes and set the stage for the evolution of a powerful signalling system: influx of calcium and membrane depolarization became the functional core of the later evolving nervous systems.

We reason here that the key signalling role of calcium-triggered depolarization in neuron and muscle physiology (where it controls, respectively, secretion and contraction), or depolarization–contraction–secretion (DCS) coupling, evolved from an ancient ‘emergency response’ to external calcium influx after membrane damage. We detail how controlled membrane depolarization and action potentials evolved from ancient voltage regulation mechanisms, and how they became coupled to the downstream responses such as ciliary beating and whole-cell contraction. Differential distribution of these functions among distinct cell types by division of labour finally gave rise to the configuration of modern neuronal and neuromuscular circuits in animals.

## 2. From membrane rupture to depolarization–contraction–secretion coupling

**Figure 1:**
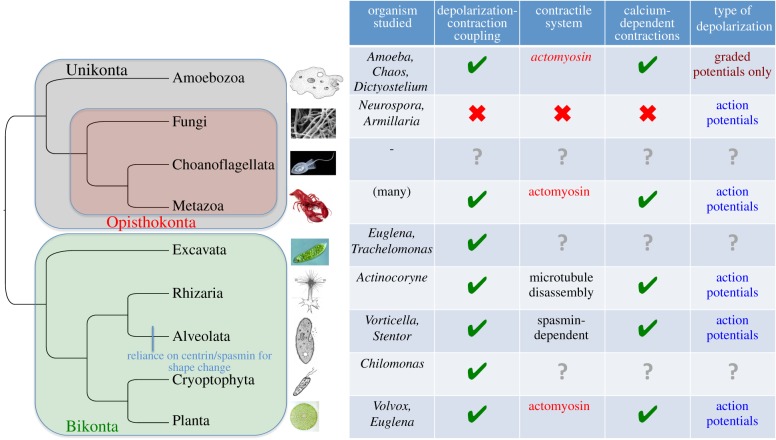
Excitation–contraction coupling across the eukaryotic tree of life. A first split between the plant lineage (Bikonta) and the animal lineage (Unikonta) is favoured by most authors [30], and we follow this view here. However, alternatives are still not excluded [31]. Data on eukaryotic groups are from the literature ([32–35] and references in the text). Green ticks indicate the presence and red crosses indicates the absence. This distribution is consistent with the presence of depolarization–contraction coupling via calcium in the last common eukaryotic ancestor.

The control of actomyosin contraction by an increase in intracellular calcium concentration, pivotal in animal muscle physiology [28], appears to be an ancestral feature of eukaryotic cells [29] (figure 1). Actin, myosin and calmodulin are virtually universally present in eukaryotic genomes [36–39]. Myosins are composed of a heavy chain containing the motor domain (with ATPase and actin-binding activities) and usually a light-chain binding neck domain. In most myosin families, the light chains are calmodulin proteins; in others, specialized calmodulin-related proteins have evolved—such as the essential and regulatory light chains of myosin II (MELC and MRLC) [40,41]. In all cases, the light chains contain an EF-hand calcium-binding domain [42–44]. (Notably, the control of contraction by direct binding of calcium to the myosin light chain is lost in vertebrates [45,46]). Besides animals, myosin-mediated cell contractions have been observed in amoebozoans [47–49] and in the green algae *Volvox* [50] and *Chara* [51]. How did this tight and ancient coupling between calcium influx and actomyosin-based contraction originate?

### (a) Local contraction and secretion originated as a damage response to uncontrolled calcium influx

Calcium concentration is always much larger (usually about 10^5^-fold higher) in the extracellular medium than in the cytoplasm. Intracellular Ca^2+^ concentration has to be maintained within a narrow margin because of the high toxicity of calcium ions (see above). Because of this extreme concentration difference, calcium is by far the ion with the steepest electrochemical gradient across the membrane (table 1).

**Table 1.** Electrochemical gradients for the main ions present in extracellular fluids. Values for human kidney cells. Adapted from Lang *et al.* [52].

ion	electrochemical gradient (mV)
Ca^2+^	−190
Na^+^	−130
CO_3_^2−^	−50
Cl^−^	−20
K^+^	+10

**Figure 2.**
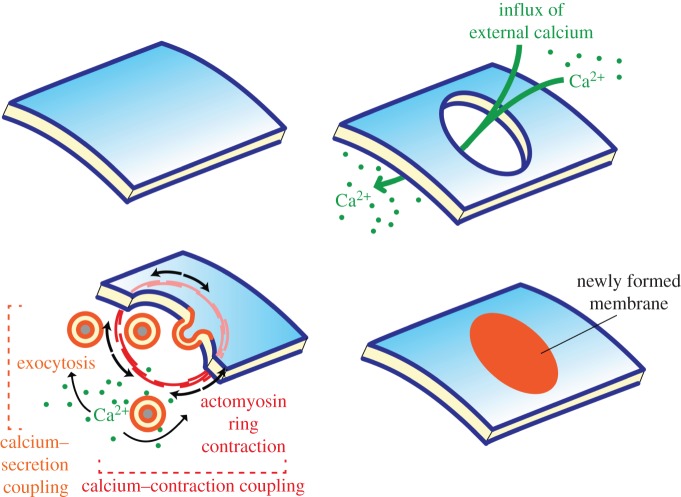
Excitation–secretion and excitation–contraction coupling in response to external Ca^2+^ during membrane repair.

Owing to this strong gradient and to its extreme toxicity, an influx of extracellular calcium within the cell is both the first detectable consequence and the main hazard of local membrane rupture. It is thus unsurprising that, in all eukaryotic cells studied, ‘wound healing’, i.e. membrane repair mechanisms are quickly activated upon local rupture, and are directly downstream of calcium ions [53]. Two major responses are conserved across eukaryotes (figure 2): (i) *contraction of an actomyosin ring around the puncture*, observed in both animals [54–57] and plants [51,58–62]; (ii) *exocytosis of vesicles that seal the damaged membrane*. The latter response is directly triggered by calcium activation of SNAP-25 and synaptotagmin, in a striking parallel to the mechanisms of neurotransmitter release [63]. More specialized calcium-dependent proteins that control vesicle fusion in both membrane repair and neurotransmitter secretion were discovered later, such as ferlins (involved in auditory neurotransmitter secretion [64]) and annexins (involved in catecholamine secretion by chromaffin cells [65]). Membrane repair by exocytosis is observed in animals [66,67] and in plants [68–70].

We propose that this wound healing response dates back to the last eukaryotic common ancestor (LECA) and was the first manifestation of a tight coupling of depolarization (through uncontrolled calcium influx), contraction and secretion, referred to here as DCS coupling (figure 2). Membrane wound healing is a vital necessity for any eukaryotic cell which lacks a protective extracellular cell wall (as was the case of ancestral eukaryotes [71]). There must thus have existed a strong selective pressure for the evolution of membrane repair from the very first stages of eukaryotic evolution onwards. Owing to its steep concentration gradient and high toxicity, there are good reasons for calcium in particular to be the wounding signal—rather than any other ion or molecule. Finally, calcium has remained the key trigger for actomyosin contractility and exocytosis in other functional contexts, including muscle contraction; in these more specialized cases, specific mechanisms are required for calcium influx or release (from the extracellular medium or internal stores) instead of calcium influx being passively forced by wounding [72]. The general control of exocytosis by calcium release has indeed been confirmed in both plants and animals [73–75].

### (b) Anticipating damage: evolution of mechanosensitive Ca^2+^ channels

**Figure 3.**
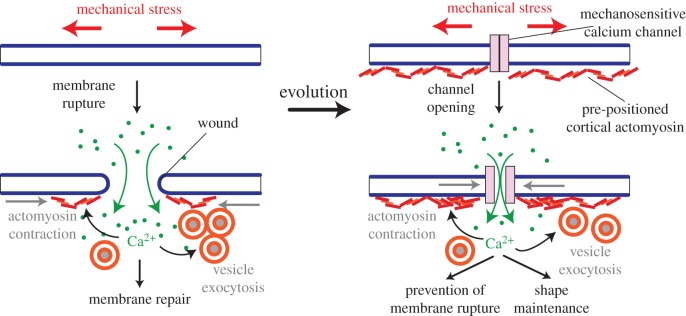
Emergence of mechanosensitive Ca^2+^ channels and cortical actomyosin for anticipating membrane damage in stem eukaryotes.

We propose that the next step in the evolution of eukaryote DCS coupling has been the recruitment of stretch-sensitive calcium channels, which allow controlled influx of calcium upon mechanical stress before the actual damage occurs, and thus anticipate the effects of membrane rupture (figure 3). Indeed, ion channels of the TRP and Piezo families known to be mechanosensitive in animals were ancestrally present in eukaryotes, and all characterized members are either partly or uniquely calcium-permeant [76–79]. The mechanosensitive role of Trp channels has been demonstrated both in animals [78] and in the green alga *Chlamydomonas* [80], while bikont Piezo channels still await functional characterization—but mechanosensitive calcium incurrents (by mostly unknown channels) are broadly present in plants [81,82].

To prevent the actual rupture, the first role of mechanosensory Ca^2+^ channels might have been to pre-activate components of the repair pathway in stretched membranes. As another anticipatory step, actomyosin might have been pre-positioned under the plasma membrane (hence the cortical actomyosin network detected in every eukaryotic cell) and might have also evolved direct sensitivity to stretch: the ATPase activity of myosin is stimulated by tension via the small GTPase Rho and the ROCK kinase [83], which are also active in membrane repair [84]. Once its cortical position and mechanosensitivity were acquired, the actomyosin network could automatically fulfil an additional function: cell-shape maintenance—as any localized cell deformation would stretch the cortical actomyosin network and trigger an immediate compensatory contraction (figure 3). This property would have arisen as a side-effect (a ‘spandrel’ [85]) of the presence of cortical actomyosin for membrane repair, and quickly proved advantageous.

### (c) Evolution of amoeboid movement

**Figure 4.**
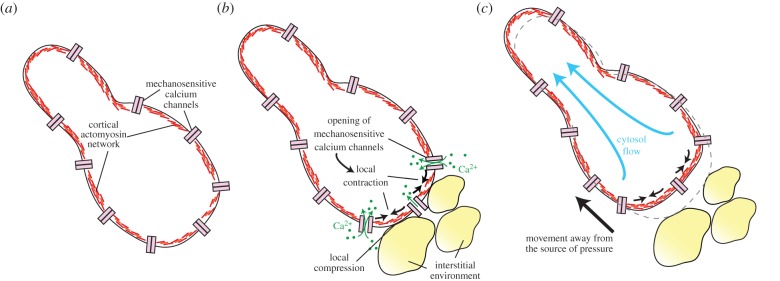
A hypothesis on the origin of proto-amoeboid movement as a mechanically induced escape response. (*a*) Cortical actomyosin and mechanosensitive Ca^2+^ channels were probably present in stem eukaryotes. (*b*) Localized compression, for example, in an interstitial environment, would have triggered channel opening and local contraction of the cortex. (*c*) Local cell contraction would have mediated escape from the source of pressure. Such a simple escape response, building on ancient eukaryotic modules, could have been the first manifestation of a simple ancestral proto-amoeboid movement.

Once covering the cell cortex, the actomyosin network acquired the ability to deform the cell by localized contraction. Actomyosin-mediated cell deformation is especially instrumental in amoeboid locomotion, in which part of the cytoplasm undergoes pulsatile contraction that project the rest of the cell forward. Based on the genomic study of the protist *Naegleria* [86], which has a biphasic life cycle (alternating between an amoeboid and a flagellated phase), amoeboid locomotion has been proposed to be ancestral for eukaryotes. It might have evolved in confined interstitial environments, as it is particularly instrumental for cells which need to move through small, irregularly shaped spaces by exploratory deformation [87]. Amoeboid locomotion has recently been the focus of regained interest with the discovery that a surprisingly wide diversity of animal cell types (both embryonic and adult) can undergo a switch to fast amoeboid locomotion under high-confinement, low-adhesion conditions [88,89]. This ‘amoeboid switch’ has been speculated to be evolutionarily ancient [88], and might recapitulate an ancestral protist escape response to pressure. One can hypothesize that, if stretch-sensitive calcium channels and cortical actomyosin were part of the ancestral eukaryotic molecular toolkit (as comparative genomics indicates), membrane deformation in a confined environment would probably trigger calcium influx by opening of stretch-sensitive channels, which would in turn induce broad actomyosin contraction across the deformed part of the cell cortex, global deformation and cell movement away from the source of pressure (figure 4). Similarly, in migrating fish keratinocytes, stretching of part of the cell opens mechanosensitive calcium channels and triggers local cell retraction, possibly by actomyosin contraction [90]. In *Amoeba*, cell contraction has been proposed to be controlled by local cell depolarization [91,92] and calcium influx [93,94]. One can thus propose that a simple ancestral form of amoeboid movement evolved as a natural consequence of the scenario outlined above for the origin of cortical actomyosin and the calcium–contraction coupling (figure 4); once established, it could have been further elaborated. As a note of caution, the molecular mechanisms that mediate the amoeboid switch under pressure are still unknown. If they involved mechanotransduction by calcium influx, for example, via stretch-sensitive calcium channels, this would support our evolutionary hypothesis. Direct stretch-sensitivity of the actomyosin network (for example via ROCK) might also have contributed.

### (d) The control of flagellar beating by calcium

In addition to the actomyosin-based effector system, the LECA also possessed microtubule-based cilia [95–97]. These were both sensory and motile, representing an independent sensory–effector system in a separate cellular compartment. Besides cAMP and cGMP, calcium plays a conserved role in the control of ciliary beating [98]. It is thus tempting to speculate that, once calcium signalling had gained control over primitive forms of amoeboid movement, the same signalling system started to modify ciliary beating, possibly for ‘switching’ between locomotor states. In *Naegleria*, calcium signalling stabilizes the amoeboid phase at the expense of the flagellated phase [99,100]. If calcium-induced ciliary arrest is ancestral for eukaryotes (which remains to be fully tested, see below), this might have been part of a calcium-mediated switch to amoeboid locomotion.

Calcium has a ubiquitous connection to flagellar/ciliary beating, and it is tempting to hypothesize that cross-talks between the incipient calcium signalling pathways and flagellar control were established early in evolution. However, the effects of calcium on cilia are highly taxa-specific and apparently fast-evolving, making ancestral reconstructions challenging.

In animals, calcium usually inhibits flagellar or ciliary motility: calcium induces ciliary arrest in mussel gill cilia [101–103], in ascidian gill slits [104] and in embryonic epidermal cilia of sea urchins [105]. In sperm cells of ascidians [106], sea urchins [107,108], siphonophores [109] and snails [110], calcium bursts increase the asymmetry of flagellar beating and the swimming curvature, which serves to change direction during chemotaxis [111]; in *Ciona* sperm cells, the calcium sensor has been shown to be calaxin, a protein that directly inhibits outer-arm dyneins, thus triggering beating asymmetry [112]—showing that the response of sperm flagellar beating to calcium is inhibitory at the molecular level. Exceptions are known in vertebrates, such as the cilia of the vertebrate foregut (mammalian airways and frog oesophagus [98,113]) or the flagellum of mammalian spermatozoa [114], which respond to calcium by increasing beating frequency. Another unique situation is known in ctenophores, where calcium induces ciliary reversal [115].

In other eukaryotes, calcium usually mediates a switch in the modalities of flagellar beating, but the details vary between groups. In the green alga *Chlamydomonas*, calcium induces a switch from asymmetric to symmetric beating [116,117]—thus opposite to its effect in animal sperm. Confusingly, in two other green algae—*Pterosperma* and *Cymbomonas*—calcium induces an asymmetric-to-symmetric switch, similar to animal sperm [118]. Sperm chemotaxis in the fungus *Allomyces* [119] and the brown alga *Ectocarpus* [120] requires calcium influx, like in metazoans, but it is unknown whether the mechanisms are comparable. Like ctenophores, *Paramecium* undergoes ciliary reversal in response to calcium [121]. In the trypanosome *Crithidia*, calcium induces a switch in the direction of flagellar wave propagation, from tip-to-base (a trypanosome-specific propagation mode) to the (more canonical) base-to-tip direction [122]. The molecules involved, when known, are equally disparate: the calcium sensor of the *Ciona* sperm flagellum, calaxin, is opisthokont-specific; conversely, the calcium sensor of *Chlamydomonas*, the light chain 4 of outer-arm dynein (LC4), is absent from opisthokont genomes [123].

This diversity of effects and mechanisms suggests that the ciliary response to calcium is relatively fast-evolving, which makes it difficult to deduce which effect (if any) calcium had on ciliary beating in the LECA. Possibly, in ancestral eukaryotes calcium induced a relatively simple switch (such as ciliary arrest, as still seen in many animal cells and in *Chlamydomonas* in response to high Ca^2+^ concentrations [116]), which was then gradually modified into more subtle modulations of beating mode with a fast turnover of molecular actors mediated by differential addition, complementation and loss. Alternatively, control of cilia by calcium could have evolved convergently—but such convergence would then have been remarkably ubiquitous, as there seems to be no eukaryotic flagellum that is not controlled by calcium in one way or another. Testing these hypotheses will require better mechanistic understanding of ciliary control in the taxa already studied, as well as broader taxonomic sampling, for example including *Naegleria*, flagellated amoebozoa (such as *Pelomyxa* or *Phalansterium*) or flagellated fungi (Chytridiomycota).

## 3. The ciliary origin of action potentials

*Ab initio*, membrane depolarization by calcium influx was a gradual process. In excitable cells, however, the initial membrane depolarization is not immediately followed by homeostatic return to the setpoint; rather, depolarization is first actively amplified if it goes beyond a certain threshold, and then quickly terminated. This set of events is called an action potential. Action potentials are all-or-nothing electrical spiking events, which propagate in a regenerative and unidirectional fashion across the cellular membrane (or across the membrane segment that expresses the necessary channels, unidirectionality being due to channel inactivation)—thus allowing fast concerted responses to external signals. We propose here that the first context where this enhancement/binarization of depolarization evolved was the cilium.

### (a) Evolution of depolarization-activated calcium and sodium channels

**Figure 5.**
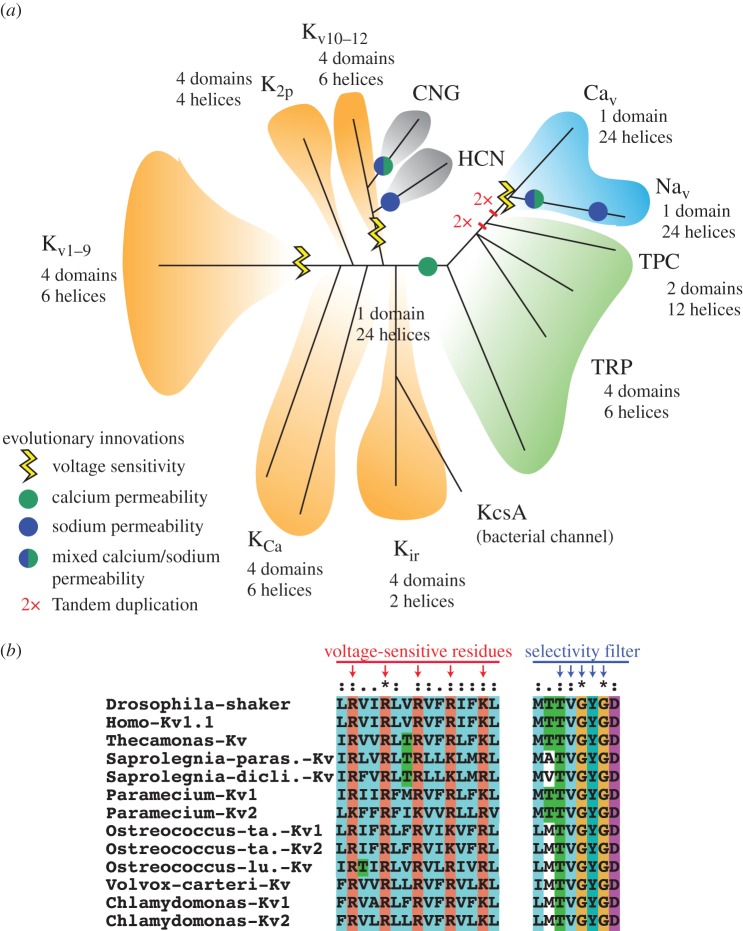
(*a*) Phylogenetic trees of voltage-gated-like ionic channels. Tree follows Yu *et al.* [137] with the added hypothesis of a sister-group relationship between TPC and Na_v_/Ca_v_ channels, as suggested by the domain structure [139,142]. (*b*) Putative K_v_ channels in bikonts and apusozoans. Putative orthologues have been identified as mutual best BLAST hits with the human K_v_1.1 sequence in a search against all eukaryotic genomes deposited in NCBI (http://blast.ncbi.nlm.nih.gov), excluding the taxa Opisthokonta and Amoebozoa. Candidates shown here belong to the genomes of the apusozoan *Thecamonas trahens* (belonging to the sister-group of opisthokonts) and the following dikonts: the oomycete *Saprolegnia* (two species: *diclina* and *parasitica*), the ciliate *Paramecium* and the green algae *Volvox*, *Chlamydomonas* and *Ostrococcus* (two species: *lucimarinus* and *tauri*). Voltage-sensitive residues (positively charged residues in every third position of the helix S4; red arrows) and the K^+^ selectivity sequence are shown as of Moran *et al.* [128]. K_v_ candidates could not be found in embryophyte genomes (apart from one sequence in the barley *Hordeum*, which might have been acquired by horizontal transfer and, due to its isolated nature, was not considered further).

Active amplification of depolarization requires the opening of voltage-gated channels permeant to external cations. The evolution of these channels was a prerequisite to the evolution of action potentials and, importantly, predated the LECA (box 1). The phylogenetic tree of voltage-gated-like ionic channels suggests that Na^+^- or Ca^2+^-permeant channels evolved by modification of the ancestral stock of voltage-buffering K^+^ channels, and that such modification happened twice [128,143,144]: one lineage gave rise to the CNG and HCN families and another led to the emergence of the Trp channels, a family of mostly thermo- or mechanosensitive and calcium-permeant channels. Importantly, Trp channels represent the sister clade to the voltage-gated sodium and calcium channels (Na_v_ and Ca_v_) that are key to the generation of action potentials figure 5*a*). Na_v_/Ca_v_ channels have been identified in genomes of choanoflagellates [145], apusozoans (the sister-group of opisthokonts) [146] and several bikont lineages [147], which makes a strong case that they existed in the LECA. The ancestral presence of voltage-gated Na_v_/Ca_v_ channels suggests that the LECA was able to support bona fide action potentials (box 1).

Box 1. **Evolution of voltage-gated ion channels**

All transmembrane proteins of the VGL (voltage-gated-like) superfamily contain a central pore delimited by four identical domains. In the ancestral state, these four domains are made of distinct polypeptidic chains, and the channel thus forms by assembly of four subunits. It is assumed that the first ion channels were probably potassium-permeant channels of the Kir type (four subunits of a very simple structure: two transmembrane domains each). Indeed, similar channels are widespread in bacteria (KcsA) [124] (figure 5*a*). Kir channels are ubiquitously expressed and respond to hyperpolarization by allowing potassium influx—so favouring reversal to the resting potential [125]. Their voltage sensitivity appears indirect and due to voltage-dependent gating by Mg^2+^ and polyamines [126,127]. The phylogenetic tree of voltage-gated-like channels suggests that, from these ancestral proteins, K^+^ channels with a more complex structures (four subunits of six transmembrane helices each) evolved, with direct voltage sensitivity (K_v_) or sensitivity to calcium influx (K_Ca_). Another branch led to the constitutively active and mechanosensitive channels of the two-pores K2P family (four transmembrane domains). K_v_ channels might predate eukaryotes, as candidate K_v_ channels are broadly detected in the genomes of both unikonts [128] and bikonts (figure 5*b*)—as had long been assumed from electrophysiological evidence of voltage-dependent K^+^ currents in plants and protists [129–131]. Moreover, similar (and possibly homologous) voltage-dependent potassium channels have been found in prokaryotes [132,133]. It is likely that all these potassium channels ancestrally contributed to the same role: maintaining the resting potential and restoring it upon accidental or controlled depolarization (as in response to membrane damage or sensory calcium influx). This is indeed still the function of K_v_ channels in non-excitable cells such as lymphocytes [134]. The sister-group of one K_v_ subfamily (K_v10–12_) is a clade of influx cationic channels that acquired sodium/calcium permeability and gating by cyclic nucleotides or hyperpolarization (CNG and HCN families) [135–137].

Sensory Trp channels have been reported in *Chlamydomonas* [80,138] and one Piezo channel (of unknown function) is present in *Arabidopsis* [82], suggesting that their emergence predates the LECA.

The Na_v_ and Ca_v_ channels acquired a peculiar one-domain structure (with all four domains joined into one unique polypeptidic chains instead of being distinct subunits) and have been proposed to be most closely related to the TPC family, a subset of Trp-like channels with an intermediate two-domain structure (thus suggesting a two-step tandem duplication history) [139]. (One voltage-dependent sodium channel detected in some bacteria, NaChBac, has a one-domain structure and might have evolved convergently to its eukaryotic equivalent [140,141]).

The ancestral Na_v_/Ca_v_ channels were probably predominantly permeable to calcium [146,148], and functioned to amplify and propagate calcium influx upon excitation. Sodium permeability, once evolved, allowed spatial segregation of sodium and calcium channels: the sodium-permeant membrane portion specialized in propagating the signal (without undergoing the toxic and/or signalling effects of calcium), while the calcium-permeant portion specialized in responding. This is observed in bilaterian neurons, with Na_v_ channels being preferentially localized along the axon and Ca_v_ channels being preferentially localized in the pre-synaptic active zone [149,150]. The Na_v_ and Ca_v_ families diverged before the last common ancestor of opisthokonts, as both can be detected in apusozoan genomes, together with mixed identity Na_v_/Ca_v_ channels apparently basal to the split [146]. Early Na_v_ family members were permeant to both sodium and calcium, and full specificity to sodium only evolved in bilaterians and in medusozoans, probably in line with more active lifestyles and faster movements [145].

### (b) The first action potentials and their function

Once Na_v_ and Ca_v_ channels existed, action potentials were possible, and could spread in a regenerative fashion over the domain of the plasma membrane that contains these channels. Action potentials allow a fast, global binary response to depolarization over the whole membrane domain that expresses the right channel complement.

What could have been the ancestral function of action potentials? While amoeboid movement is probably ancient (as argued above), electrophysiological recordings of amoebae only indicate the involvement of graded potentials [91,151,152]—unsurprisingly, as amoeboid movement relies on (graded) contractions of part of the cortex rather than a global binary response. Some cell-wide binary responses mediated by action potentials have been described in other protists—for example, the escape response of *Stentor*, *Vorticella* and *Actinocoryne* (box 2)—but they involve highly specialized mechanisms and are probably derived. Action potentials of unknown function have also been detected in the green algae *Chara* [173], *Eremosphaera* [174,175] and *Acetabularia* [51], and in the diatom *Odontella* [176].

Box 2. **Specialized calcium-controlled contractile systems.**

In some eukaryotic lineages, the ancestral actomyosin system was complemented or replaced by other, more taxonomically restricted contractile modules that allowed even faster contractions. These are instrumental, for example, in escape response. Notably, in all known cases, control by calcium appears to have been retained. For example, many unicellular green algae, such as *Platymonas*, display a calcium-sensitive contractile protein, called centrin, as part of their striated ciliary rootlet, which, upon membrane depolarization and calcium influx, drives local membrane bending and flagellum protrusion/retraction [153–156]. In Alveolata (a group which includes ciliates and dinoflagellates), the centrin-based system is hugely expanded into a cell-wide contractile apparatus, which is able to drive contraction of the entire cell in some ciliates (like *Paramecium* or *Stentor*), or of large specialized cellular structures—such as the piston of the dinoflagellate *Erythropsidinium* [157], or the stalk of the ciliate *Vorticella* [158] which quickly retracts upon mechanical or photic stimulation. In fact, centrin has been discovered independently as part of the contractile apparatus of *Vorticella*, the spasmoneme, and given the name spasmin [34,159–166]. Another mechanism for excitation- and calcium-dependent fast retraction evolved convergently in the rhizarian *Actinocoryne* and is mediated by catastrophic disassembly of microtubules [167–172]. Both the centrin/spasmin system and the microtubule disassembly-based systems allow faster contractions during escape response than actomyosin. Both of these new mechanisms retained dependency on calcium, which might have facilitated continuous and stepwise complementation, and ultimately replacement, of actomyosin systems by these specialized mechanisms.

An interesting situation has been described in the green alga *Chlamydomonas*, where action potentials are exclusively detected in the flagellum, while the rest of the cell presents only graded potentials [177,178]. Here, action potentials mediate a fast switch in flagellar beating. The *Chlamydomonas* voltage-dependent calcium-channel Ca_v_ 2 is restricted to the tip of the flagellum [179], while the mechanosensory TRP11 channel is present at its base [138]. This peculiar organization explains the restriction of action potentials to the flagellum in *Chlamydomonas* and provides functional insights into the organization of the single-celled sensory–effector arc: external mechanical signals are detected at the flagellar base (where active bending is restricted), and action potentials spread quickly along the whole flagellum (but not the rest of the cell) to allow fast and coordinated beating reversal upon stimulation.

**Figure 6.**
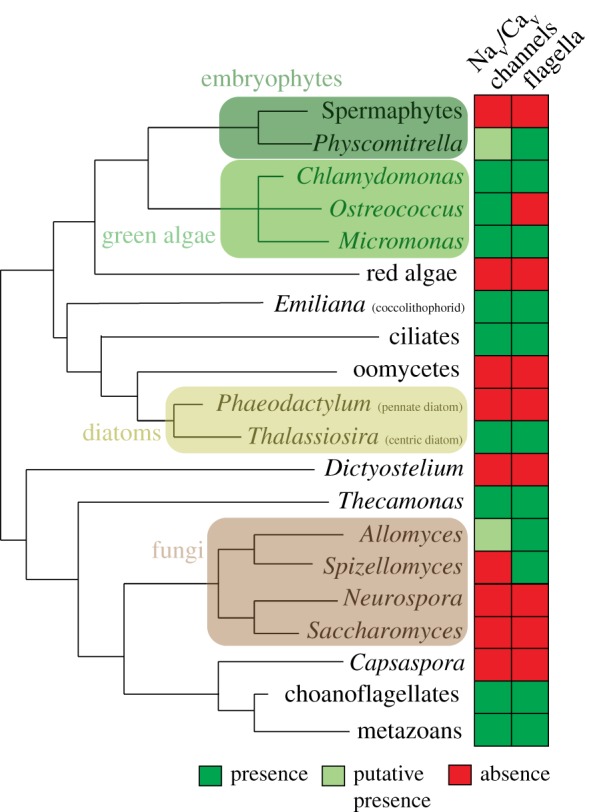
Loss of Na_v_/Ca_v_ channels correlates with loss of flagella in eukaryotes. Channel presence/absence is indicated following Moran *et al.* [128] and Verret *et al*. [147]. Putative Na_v_/Ca_v_ were identified by mutual best BLAST hits in the genomes of *Allomyces* (genome on the Broad Institute website http://www.broadinstitute.org/annotation/genome/multicellularity_project/Blast.html) and *Physcomitrella* (genome on the NCBI website http://blast.ncbi.nlm.nih.gov); no such candidate was found in spermaphyte genomes. The correlation closely follows the pattern of flagellum loss within groups such as diatoms and embryophytes. Two exceptions are *Ostreococcus* (which might have lost its flagella fairly recently, as even closely related green algae retained them [182]), and the chytridiomycete *Spizellomyces*, which has flagellated zoospores. It will be interesting to investigate the existence and determine the mechanism of flagellar beating control in the absence of Ca_v_ channels in this fungus. The CCH1 channel of yeast was originally considered a Ca_v_ homologue [183] but has been shown to be orthologous to the sodium leak channels NALCN (belonging to a branch that diverged at the base of the Na_v_/Ca_v_ clade) [184]. As a caveat, similarly detailed orthology analyses still have to be performed for bikont channels.

Several lines of evidence suggest that action potentials—and the corresponding Na_v_ and Ca_v_ channels—evolved in the context of the flagellum. First, in *Paramecium* as in *Chlamydomonas*, Ca_v_ channels are exclusively detected within the membrane of the cilia [180,181]. Second, and most important, loss of Na_v_/Ca_v_ channel has been prevalent during eukaryotic evolution—and almost perfectly correlates with cases of secondary loss of flagella (figure 6). This strongly suggests that flagella are the primary locus of action potentials in most protists. (Note that electrophysiological recordings in some ciliated or flagellated protists, such as *Paramecium* (as shown in figure 1), only showed graded potentials, but that, like in *Chlamydomonas*, action potentials might be restricted to the flagellar or ciliary membrane, which has not always been recorded).

## 4. Towards the animal nervous system

We thus propose that, in ancestral eukaryotes, the cell body membrane only showed graded potentials, while action potentials were restricted to the cilia/flagella. However, in several eukaryotic lineages, regenerative propagation of action potentials has been described—for example, in protist escape responses (box 2) and in groups forming unusually large colonies or syncytia (such as fungal hyphae [32,33] and the vascular tissues of land plants [185]), where action potentials appear to be specifically involved in long-range communication. Finally, in animals, action potentials spread from the cilium to a large part (or the totality) of the electrically excitable cell (neurons and myocytes).

### (a) The birth of mechanosensory–contractile cells

When did this shift in the spreading of action potentials from flagella to the cell body occur, which was key to the evolution of animal nervous systems? Choanoflagellate electrophysiology is undescribed, and it would be interesting to know whether action potentials are restricted to their flagellum or also invade the cell body. Regarding sponges, the data is equally scarce. Interestingly, functional assays suggest that cellular sponges lack action potentials, as well as the ability to stop the flagellar beating of choanocytes [186], and K_v_ and Na_v_ channels appear lost from the *Amphimedon* genome (but some Ca_v_ are retained) [128]. This suggests that spreading of action potentials beyond the cilium may have only been acquired after the sponge lineage diverged from other animals—and that cellular sponges underwent some degree of loss of electric excitability when they lost the ability to control flagellar beating. The only exception appears to be the syncytial glass sponges, in which global arrest of flagellar beating is coordinated by action potential propagation along the syncytium [187].

In ctenophores, action potentials have been recorded from the cell bodies of the large ciliary comb cells [188] as well as muscle cells (see below); and in most eumetazoans, action potentials are likewise detected in other cell parts beyond the cilium—most prominently, the neuronal axon or the whole sarcolemma of contractile cells. This has enabled global cellular responses such as concerted contraction, representing the birth of mechanosensory–contractile cells, that act in the context of a whole-tissue contraction (rather than in a cellular context as found during amoeboid movement, see above).

**Figure 7.**
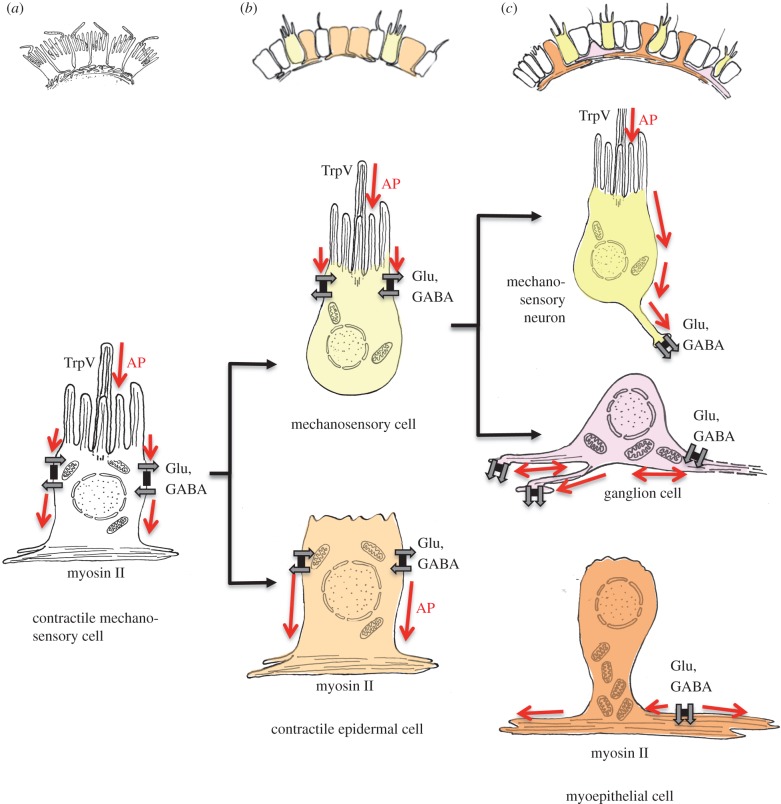
Origin of mechanosensory–contractile circuits by division of labour. (*a*) Hypothesized ancestral plurifunctional sensory–contractile myoepithelial cell. Upon activation of apically located mechanosensory TrpV channels, action potentials are generated in the cilia, propagated along the cell body and activate contraction of a basal process within the same cell. Ancient signals (such as glutamate or GABA) are secreted by lateral exocytosis and detected by neighbouring myoepithelial cells to spread the contraction wave. (*b*) The first division of labour results in the split between mechanosensory cells, specialized in signal perception and transmission, and contractile cells. Mechanosensory cells retain TrpV channels, action potentials and secretion. Contractile epidermal cells retain action potentials and contraction. (*c*) The second division of labour results in the split between mechanosensory neurons, which exclusively secrete neurotransmitters but do not respond to them, and interneurons (or ganglion cells) that respond to neurotransmitters and activate contraction of the myoepithelial cells.

How did this spread occur? A tantalizing possibility (depicted in figure 7) is that, in early mechanosensory–contractile myoepithelial cells [189], the action potential was regeneratively propagating along the whole cell—across the entire apical and basolateral membranes until it reached the basal contractile process. Such cell-wide action potentials have indeed been proposed to exist, for example, in bipolar spider mechanoreceptors, where action potentials originate in a sensory dendrite, and are propagated to the soma and the axon [190]. Alternatively, the electric signal might have been passively conducted across the soma and re-amplified in the basal contractile parts of the cell.

### (b) Evolution of neurons and myocytes by division of labour

In extant ctenophores [191], cnidarians [192] and bilaterians, action potentials have been recorded from neuronal cell types as well as diverse myocytes, corroborating the idea that muscle cells and neurons arose from mechanosensory–contractile cells by division of labour [189,193]. Following this scenario, various sensory, secretory and contractile modules and functions were segregated to different cell types, so that the depolarization–secretion and depolarization–contraction couplings became the functional core of neuron and myocyte physiology, respectively (figure 7). Action potentials would have been selectively retained where there is a need for either cell-wide all-or-none response (such as muscle contraction) or long-range propagation (such as along axons).

Myocytes specialized on converting calcium signals into contractions. In line with that specialization, additional molecular actors have evolved in animals to confer heightened calcium sensitivity to myosin—including the myosin light-chain kinase (MLCK) controlled by calmodulin (at the base of Metazoa), troponin C (in the striated muscles of bilaterians) and caldesmon (in the smooth muscles of vertebrates) [29]. In vertebrates, these upstream regulators have entirely taken over, as direct sensitivity of myosin to calcium has been lost [46]. Myosin evolution thus illustrates the frequent theme in molecular evolution of irreversible increase of complexity by evolution of redundant mechanisms within a pathway, followed by differential loss of function between its components [194].

## 5. Conclusion

A clear pattern is emerging that the complex electrical signalling mechanisms of animal neuromuscular circuits emerged from similar properties in single-celled eukaryotes, and that those ultimately derive from emergency responses to accidental events such as cell wounding. Over evolution, our cells acquired the ability to mimic these accidents, by letting in external calcium ions from the environment (or releasing them from internal stores). We argue that the choice of calcium as a ubiquitous ‘informational ion’ can be ultimately tracked down to its high toxicity, and to the necessity to exclude it from the cytoplasm.

Our scenario is testable in several important ways. The role of the DCS coupling in membrane repair should be generally conserved in eukaryotes, beyond plant and animals. Stretch-sensitive calcium channels would be expected to play a role in pressure-induced switch to amoeboid locomotion. Flagellum- or cilium-restricted action potentials, with corresponding restriction of Na_v_/Ca_v_ channels, should be present in more groups besides *Chlamydomonas* and *Paramecium*. Increased taxonomic sampling should continue to reveal calcium-regulated switches in flagellar or ciliary beating across eukaryotes, and might help to determine the ancestral nature of this switch.
